# Molecular and Functional Characterization of H_v_1 Proton Channel in Human Granulocytes

**DOI:** 10.1371/journal.pone.0014081

**Published:** 2010-11-23

**Authors:** Gábor L. Petheő, Anna Orient, Mónika Baráth, István Kovács, Bence Réthi, Árpád Lányi, Anikó Rajki, Éva Rajnavölgyi, Miklós Geiszt

**Affiliations:** 1 Department of Physiology, Faculty of Medicine, Semmelweis University, Budapest, Hungary; 2 Laboratory of Neurobiochemistry and Molecular Physiology, Hungarian Academy of Sciences, Budapest, Hungary; 3 Institute of Immunology, Medical and Health Science Center, University of Debrecen, Debrecen, Hungary; National Institute of Environmental Health Sciences, United States of America

## Abstract

Voltage-gated proton current (I_Hv_) has been characterized in several cell types, but the majority of the data was collected in phagocytes, especially in human granulocytes. The prevailing view about the role of I_Hv_ in phagocytes is that it is an essential supporter of the intense and sustained activity of Nox2 (the core enzyme of the phagocyte NADPH oxidase complex) during respiratory burst. Recently H_v_1, a voltage-gated proton channel, was cloned, and leukocytes from H_v_1 knockout mice display impaired respiratory burst. On the other hand, hardly anything is known about H_v_1 in human granulocytes. Using qPCR and a self made antibody, we detected a significant amount of H_v_1 in human eosinophil and neutrophil granulocytes and in PLB-985 leukemia cells. Using different crosslinking agents and detergents in reducing and non-reducing PAGE, significant expression of H_v_1 homodimers, but not that of higher-order multimers, could be detected in granulocytes. Results of subcellular fractionation and confocal imaging indicate that H_v_1 is resident in both plasmalemmal and granular membrane compartments of resting neutrophils. Furthermore, it is also demonstrated that H_v_1 accumulates in phagosome wall during zymosan engulfment together with, but independently of Nox2. During granulocytic differentiation early and parallel upregulation of H_v_1 and Nox2 expression was observed in PLB-985 cells. The upregulation of H_v_1 or Nox2 expression did not require the normal expression of the other molecule. Using RNA interference, we obtained strong correlation between H_v_1 expression and I_Hv_ density in PLB-985 cells. It is also demonstrated that a massive reduction in H_v_1 expression can limit the Nox2 mediated superoxide production of PLB-985 granulocytes. In summary, beside monomers native H_v_1 forms stable proton channel dimer in resting and activated human granulocytes. The expression pattern of H_v_1 in granulocytes is optimized to support intense NADPH oxidase activity.

## Introduction

Voltage-gated (depolarization-activated) proton current (I_Hv_) has been described in a set of mammalian and non-mammalian cells. Most studies characterizing the biophysical and pharmacological properties of this current have been conducted on human cells of hemopoietic origin, such as macrophages, lymphocytes, leukemia cell lines and granulocytes (for a detailed review on I_Hv_s see: Ref. [1]). The identity of the I_Hv_ carrying molecule had been obscure for many years, but in 2006 two groups have cloned a novel “voltage sensor only protein” (VSOP) from mouse [2] and human [3]. Heterologous expression of the two mammalian VSOPs induced the emergence of characteristic voltage-gated proton currents in a variety of cell lines [2]–[4]. Based on these results the name Hydrogen Voltage-gated Channel 1 was coined, and now is widely used to refer to the genes encoding these VSOPs (HVCN1) and to their products (H_v_1). Importantly, purified and reconstituted human H_v_1 induced depolarization-dependent proton permeability in liposomes [5], ultimately proving that H_v_1 can function as a depolarization-activated proton pathway. A series of publications have also demonstrated that mouse and human H_v_1, although functional in the monomeric form, tend to form dimers in transfected cells [6]–[8].

Despite the extensive studies, little is known about the function of the voltage-gated proton channel in leukocytes and in other cell types [9]. In case of phagocytes the widely accepted view is that this channel is essential to blunt the potentially deleterious consequences of phagocyte NADPH oxidase (phox) activity. Upon activation the core enzyme of the phox complex Nox2 (a.k.a. gp91*^phox^*) transports electrons from NADPH to molecular oxygen to produce superoxide. Superoxide anion is then converted into other, more toxic reactive oxygen species (ROS) which are involved in killing pathogens [10]. Without compensatory mechanisms the intensity of the transmembrane electron transport is high enough to generate intolerable membrane depolarization and intracellular acidification, which would eventually also block the oxidase itself [11]. Supporting the role of H_v_1-mediated proton flux in charge compensation, it was shown recently that leukocytes from H_v_1 knockout (H_v_1^−/−^) mice display 30 to 75% reduction in phorbol ester-induced ROS production [12]–[14]. Importantly, H_v_1^−/−^ cells were devoid of voltage-gated proton current [12]–[14], and their ROS production could be normalized by incorporating a proton permeable, monovalent cation channel (gramicidin) into their membrane [14]. Beside the compromised oxidase activity, neutrophils from H_v_1^−/−^ mice also displayed abnormal migration and intracellular Ca^2+^ turnover [14]. It has to be noted, however, that H_v_1^−/−^ cells also produced massive amounts of ROS [12]–[14], and an impact of H_v_1^−/−^ genotype on leukocyte development is not yet ruled out.

Results obtained using heterologous expression systems or knockout (KO) mice, although indispensable in many respects, can provide only limited and indirect information on the expression and function of H_v_1 in human cells. Therefore, amongst others, the following questions remain open concerning the proton channel molecule of human granulocytes: A) Is HVCN1 the gene that codes for proton channel in human granulocytes? B) Assuming that H_v_1 is in fact the proton channel of granulocytes, is it expressed as a monomer or multimer, and does the activation with phorbol-12-myristate-13-acetate (PMA) alter the tendency of H_v_1 to adopt these structures? C) To what cellular membranes does H_V_1 localize in resting and in phagocytosing granulocytes? D) Do H_v_1 and Nox2 colocalize in granulocytes? Importantly, data from patch-clamp studies indicated the codistribution of the proton channel with Nox2 in human eosinophils [15]. Furthermore, it has been advocated by different work groups that the proton channel is a built-in part of the phagocyte oxidase [16], [17]. E) Is normal H_v_1 expression required for normal expression and/or activity of Nox2?

To clarify the above uncertainties, we set out to define the main characteristics of H_v_1 expression in human granulocytes on the cellular, subcellular and molecular level and also its relationship to Nox2. Our results indicate that H_v_1 is expressed in intracellular membranes and on the cell surface of granulocytes. Native H_v_1 molecule is expressed in dimer and monomer form in different leukocytes and its expression is required for normal voltage-gated proton currents in different leukemia cell lines. Additionally, H_v_1 and Nox2 colocalize in granulocytes, and both accumulate in the same membrane compartment upon activation. Furthermore, severely impaired H_v_1 expression can reduce the maximum rate of superoxide release in a human granulocyte cell line PLB-985.

## Results

### Expression of H_v_1 in human leukocytes

**Figure 1:**
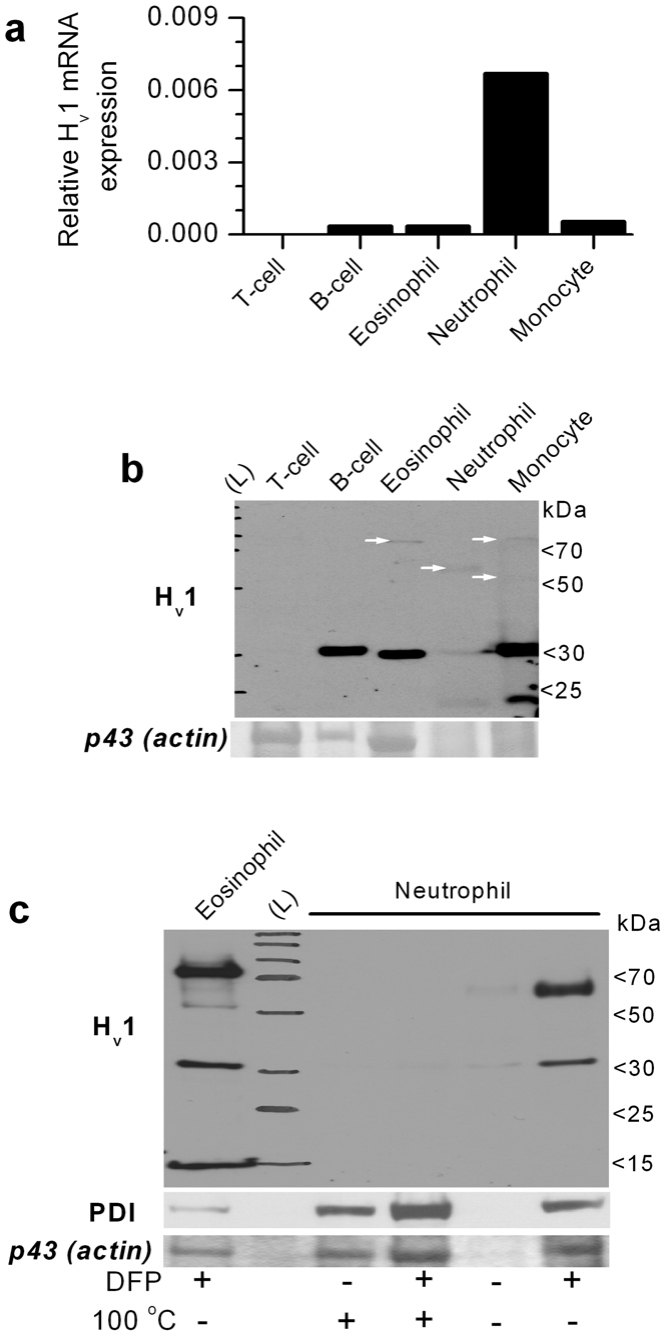
Expression pattern of H_v_1 mRNA and protein in major classes of human peripheral blood leukocytes. (**a**) Real-time qPCR analysis of H_v_1 mRNA expression relative to cyclophilin mRNA level (similar relative expressions were observed if genes other than cyclophilin were used for normalization). Data represent the mean result of a duplicate experiment. The experiment was repeated in an independent set of leukocytes with similar results. (**b**) Western blot analysis of H_v_1 expression in the same cell types as detected with aH_v_1-N. Total cell lysates of 10^6^ cells were loaded each lane. The lane with stained molecular weight marker is labeled with (L). Ponceau-stained ∼43 kDa apparent M_w_ protein band (putative actin) demonstrates the protein load and the quality of the samples. The absence of clear actin band in the neutrophil and monocyte lanes indicates massive protein degradation. Note that immunolabeling of proteins which are considered “house keeping” (e.g. actin or PDI) is of very limited use in case of cell types with very different proteolytic activity, motility and metabolism. Importantly, aH_v_1-N also detects one or more faint band between ∼60 and ∼80 kDa in myeloid cell types (white arrows). (**c**) Detection of H_v_1 and higher M_w_ bands is hampered in granulocytes by serine proteases and sample heat treatment. Total cell lysates of 5×10^5^ cells were loaded each lane. Ponceau-stained ∼43 kDa apparent M_w_ protein band (putative actin) and aPDI labeling demonstrate the degree of protein degradation. Note that anti-PDI signal and the intensity of the actin band is well correlated, which justifies the use of the latter signal to demonstrate protein load and sample quality. DFP incubation for 30 min on ice before cell lysis and heating of the sample (at 100°C for 10 min) were applied as indicated. This experiment was designed based on results from pilot studies, and was performed for demonstration purposes only, thus it was not repeated in this form. In diverse experiments low M_w_ H_v_1 labeling was occasionally observed after DFP treatment, which was independent of the granulocyte type, and likely reflects some remaining protease activity in the given sample.

We used two approaches to verify the expression of H_v_1 in human leukocytes. To assess the mRNA level, quantitative real time PCR assays (qPCR) were performed. To detect the expression of the H_v_1 protein, total cell lysates were prepared from different human blood cells. Because of their limited availability, we did not attempt to perform the experiments on basophil granulocytes and natural killer cells. We have raised an affinity purified, polyclonal antibody (aH_v_1-N) that selectively recognizes the intracellular N-terminal domain of the human H_v_1 protein in Western blots (WB) and immunofluorescence experiments (see Materiala and Methods and Fig. S1 for details). As it is demonstrated on Figs. 1a and 1b, both qPCR and WB indicated that all tested leukocyte subsets except T-cells express significant amount of H_v_1 mRNA and display significant aH_v_1-N labeling around 30 kDa. This result is in agreement with reports on the density of voltage-gated proton current in human leukocytes [1]. Similar to T-cells, membrane sample of erythrocytes lacked significant aH_v_1-N labeling in WB (not shown).

### H_v_1 forms stable dimers in human granulocytes

An interesting outcome of the WB experiment was that each of the phagocytic cell types displayed faint aH_v_1-N labeling at molecular weights significantly higher than 30 kDa, typically between 60 and 80 kDa. Furthermore, in neutrophils and monocytes a lower M_w_ band was also present. We assumed that the variability in the labeling pattern could arise from the presence of H_v_1 dimers (as described in heterologous expression systems [6]–[8]) and from proteolytic degradation of H_v_1. Granulocytes, especially neutrophils, express high amounts of various proteases, which may explain the discrepancy between the low H_v_1 protein signal and high mRNA expression level. Therefore, in follow-up experiments we attempted to improve the detection of H_v_1 and its putative dimer in granulocytes. Fig. 1c demonstrates that heat exposure and the action of DFP-sensitive serine proteases reduce the amount of detectable monomeric and high M_w_ H_v_1 forms. The detrimental effect of serine proteases could be reasonably evaded if the membrane permeable protease inhibitor DFP had been added to granulocytes before cell lysis (see Material and Methods for details). The remaining low M_w_ labeling (e.g. in the eosinophil sample in this experiment) indicates that H_v_1 degradation could not be completely prevented. Interestingly, the dominant high M_w_ band migrated with different speeds in case of eosinophils and neutrophils in this experiment. Upon repeated testing, however, no correlation between granulocyte type and migration velocity of the putative dimer could be established. Additionally, a double band around 70 kDa was observed sometimes. It is unlikely that one of the high M_w_ bands arises from a H_v_1 bound accessory protein, since Koch et al. observed similar double band migration of the heterologously overexpressed mouse H_v_1 dimer in non-reducing PAGE [8]. Therefore, we assume that the H_v_1 dimer can migrate at least in two different stable conformations.

**Figure 2:**
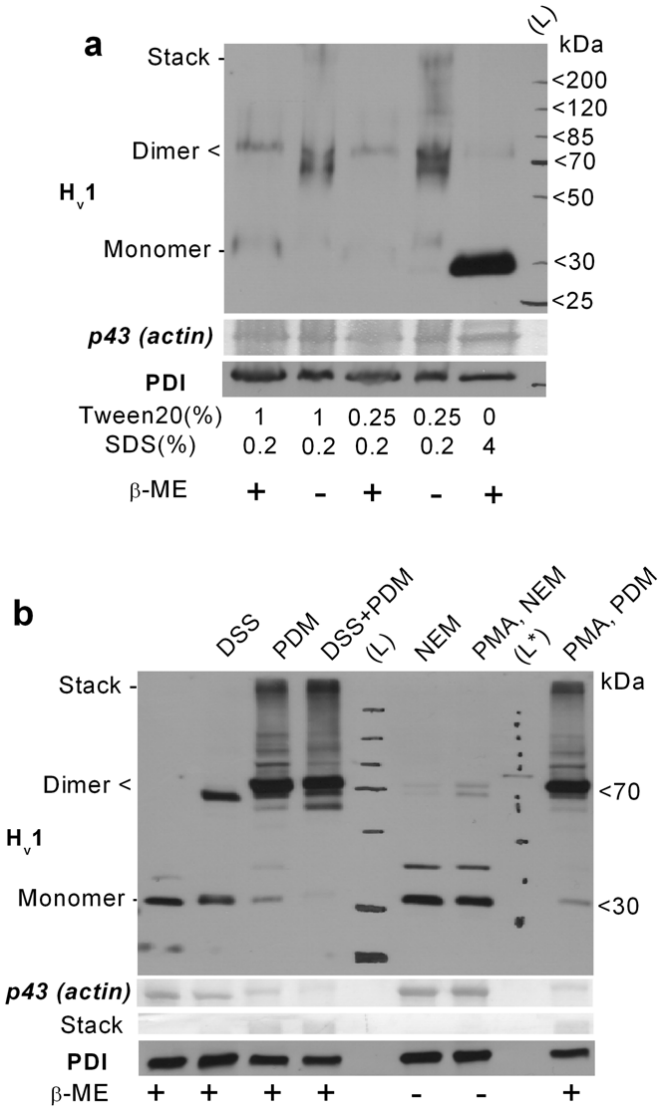
H_v_1 dimers are natively present in human granulocytes. (**a**) Total cell lysates of 10^6^ PMNs were loaded each lane. DFP-treated cells were lysed in modified 2x Laemmli sample buffer supplemented with 2 mM PMSF. Detergent composition and the presence of 5% v/v β-mercaptoethanol in the sample buffer are indicated below. (**b**) WB detection of H_v_1 dimers in granulocyte samples treated with amino- (DSS) or thiol-reactive (PDM) crosslinkers or with thiol-reactive alkylating agent (NEM). The increase in labeling with aH_v_1-N at ∼70 kDa is at the expense of that at ∼30 kDa in cross-linked samples. The appearance of higher (above 75 kDa) M_w_ aH_v_1-N-labeled bands is likely, at least in part, due to crosslinking of H_v_1 with other proteins. PMA pretreatement (200 nM for 15 min) exerted only minor effect on the vicinal cysteines in the H_v_1 dimer. The identity of the faint, ∼40 kDa band is not clear, and it was not consistently detected. Ponceau-stained ∼43 kDa band (putative actin) and near stack region demonstrates the protein load and sample crosslinking. The presence of 5% v/v β-mercaptoethanol in the sample buffer is indicated below. L* denotes unstained M_w_ marker used for calibrating the routinely used, stained M_w_ marker.

To further explore the interactions between monomers, granulocyte cell lysates were prepared using 2x Laemmli sample buffers with modified detergent compositions, and with or without the addition of the reducing agent β-mercaptoethanol (β-ME). To minimize the re-oxidation of the sample due to β-ME decomposition, it was added shortly (∼15 min) before starting the PAGE. Results in Fig. 2a indicate that the presence of β-ME and high concentration of ionic detergent (SDS) favors the detection of the H_v_1 monomer. Using mainly non-ionic detergent (Tween20) during sample preparation, the dimer form is prevailing, which migrates in two major bands under non-reducing conditions. The above data strongly indicate that H_v_1 dimers are present natively in human granulocytes and that both strong polar interactions and disulfide formation are capable for stabilizing the dimer upon solubilization.

The use of non-ionic detergent is advantageous for preserving native protein-protein interactions, e.g. to confirm the natural presence of such interactions between H_v_1 monomers. On the other hand, low SDS concentration may not denature proteases and may not provide sufficient negative charge to proteins. These drawbacks result in fainter and less clear bands in Western blot experiments, due to protein degradation and less uniform migration velocity. Therefore, in follow-up experiments we attempted to stabilize native dimers using crosslinking agents to detect them in standard SDS-PAGE. The amino group-specific bifunctional crosslinker disuccinimidyl suberate (DSS) was earlier used by two groups to improve the detection of H_v_1 dimers in H_v_1 transfected cell lines [7], [8]. Treating granulocytes with DSS before cell lysis, efficiently increased the detection of the dimeric H_v_1 form in standard reducing PAGE (Fig. 2b). Additionally, the presence of H_v_1 homodimers in all major types of human leukocytes and in differentiated PLB-985 granulocytes could be confirmed using DSS (Fig. S2b). Because monomeric and dimeric H_v_1 forms support kinetically different voltage-gated proton currents [8], it was suggested that the transition between the two forms could lead to some of the proton current phenotype changes observed in granulocytes during phox activation. [14]. Importantly, however, pretreating cells with a supramaximal phox activating dose of PMA (200 nM for 15 min) before DSS addition did not alter its crosslinking potential in granulocytes or in differentiated PLB-985 cells (data not shown).

Although H_v_1 dimers do not seem to decompose upon cell activation, disulfide formation has been reported to participate in redox regulation of different ion channels [18]. We used N-ethylmaleimide (NEM) and *N*,*N*′-(1,3-phenylene)dimaleimide (PDM) to explore the possible influence of redox changes during the PMA-induced oxidative burst on disulfide formation. Both NEM and PDM react covalently with reduced cysteine residues, but while PDM is a homobifunctional crosslinker and mimics disulfide formation, NEM prevents it. To get a snapshot on the redox state of the cysteines possibly involved in H_v_1 dimerization, the thiol reactive compounds were added after the cells were placed on ice. PDM could drive dimer formation near to completion during the 40 min incubation. On the other hand, NEM almost completely prevented the detection of the dimer independently of PMA preactivation (Fig. 2b). These results indicate that although certain cysteines in the apposed H_v_1 monomers become vicinal, they are mainly in reduced state. Massive oxidation of the apposed cysteines is unlikely even in PMA-activated cells, as the action of PDM and NEM on dimer formation was hardly affected by PMA treatment. Importantly, PMA treatment did not alter the amount of H_v_1 protein in granulocytes. The detected H_v_1 density in WBs from PMA-treated samples was 96±10% (n = 5) of that from resting cells.

It is theoretically possible that not monomer and dimer, but a higher-order multimer is the dominant native confirmation of H_v_1 in granulocytes. We reasoned that we should be able to detect such multimers as clear and dominant band if we apply more intense crosslinking (DSS + PDM for 1 h) or if we solubilize cells with non-ionic detergent after DSS crosslinking. In all cases, however, massive labeling was detected only around 70 kDa and in the stacking region, even if monomers became completely undetectable (data not shown). Labeling in the stacking region may result form non-specific interactions and/or network like crosslinking of macromolecular complexes. Therefore, if higher-order multimers are present in granulocytes their occurrence is much less likely than that of dimers or interactions with other proteins.

### H_v_1 primarily localizes to intracellular membranes in granulocytes

**Figure 3:**
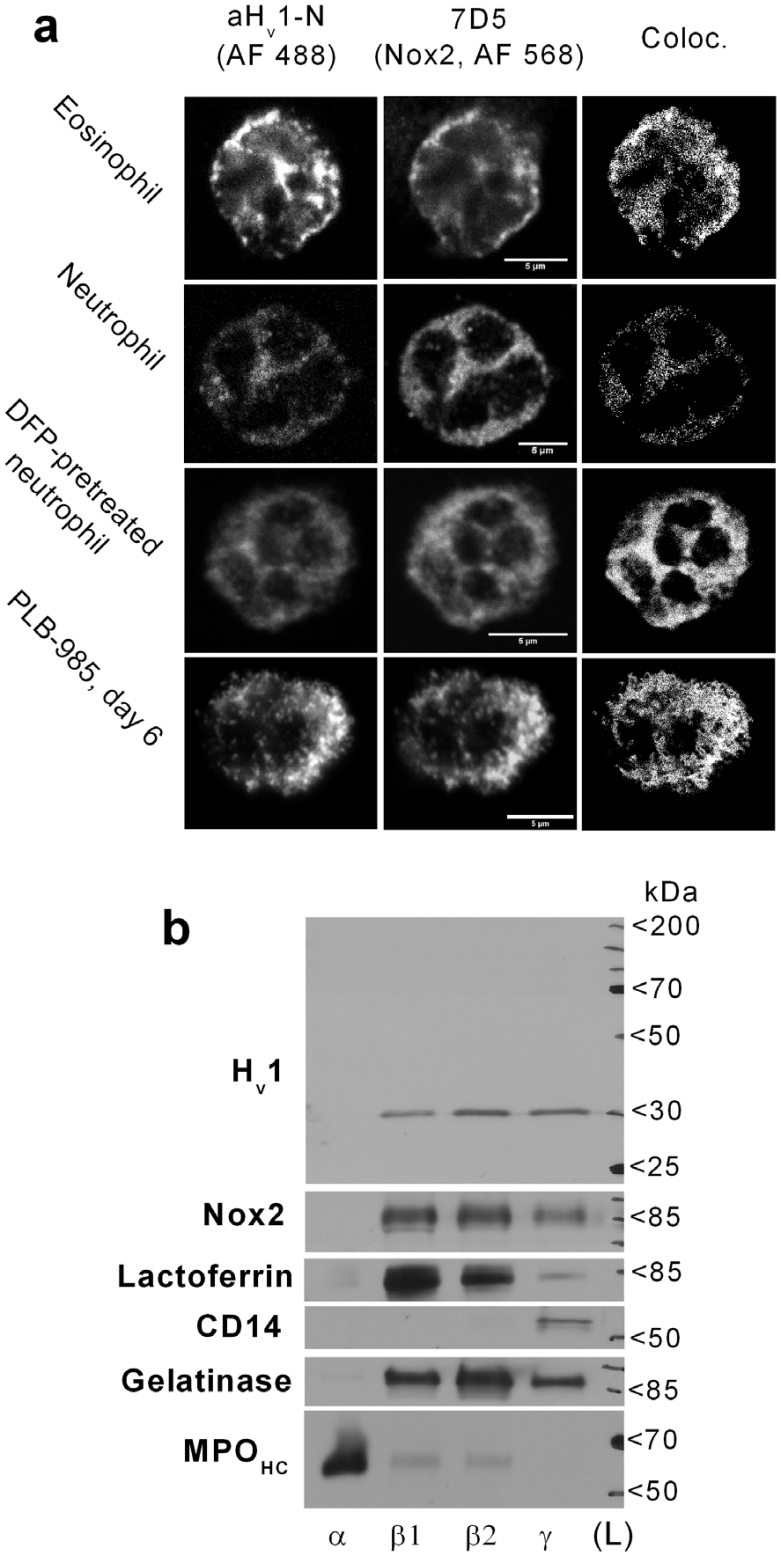
Partial colocalization of H_v_1 and Nox2 in different types of human granulocytes. (**a**) Detection of H_v_1 (left-most column) and Nox2 (middle) in granulocytes. (Scale bars represent 5 µm). Pretreating neutrophils with DFP improved the detection of H_v_1. Colocalization analysis was performed only in cells, in which above-threshold labeling (two times above background, see below) for both proteins could be detected. Colocalizing pixels are displayed as white dots in the most right column. Negligible Alexa Fluor® signals (considered as background) were detected with control primary antibodies (not shown). (**b**) Western blot analysis of the distribution of H_v_1 and Nox2 between granule fractions of resting neutrophils after standard reducing PAGE. Ponceau stain confirmed that similar protein amount was loaded each lane (not shown). The different fractions are: azurophil (α), specific (β_1_), gelatinase (β_2_) granules and secretory vesicle together with plasma membrane (γ). Immunodetection of lactoferrin, gelatinase, CD14 and the heavy chain of myeloperoxidase (MPO_HC_) demonstrates the purity of the membrane preparates [22].

Data on the subcellular distribution of native H_v_1 in human granulocytes is lacking, but a recent study demonstrates that the heterologously overexpressed H_v_1 locates to intracellular membrane compartments in HeLa cells [19]. The accumulation of H_v_1 in phagosome membrane in mouse granulocytes [12] and in late endosomes of human lymphocytes [20] was also demonstrated. To explore the subcellular distribution of H_v_1 in human granulocytes, purified, adherent granulocytes, adherent PLB-985 granulocytes and smears of whole blood were labeled with aH_v_1-N along with the monoclonal anti-Nox2 antibody 7D5 [21]. Nox2 labeling was routinely applied to see whether the strong functional coupling between Nox2 and the voltage-gated proton channel [11] is mirrored in their distribution. Another rationale of 7D5 colabeling is that the localization of Nox2 in granulocytes is relatively well explored [22], [23]. Distribution of H_v_1 and Nox2 was detected using confocal laser scanning microscopy. Eosinophils displayed strong labeling with aH_v_1-N, while neutrophils generally were labeled weaker but with variable intensity (Fig. 3a). Differentiated PLB-985 cells also displayed significant labeling for H_v_1. Subcellular colocalization of H_v_1 and Nox2 was a general finding. The lowest correlation in the two signal intensities was detected in neutrophils. Pearson's coefficients for neutrophils, eosinophils and differentiated PLB-985 cells were 0.68±0.03 (n = 5) 0.84±0.03 (n = 5), and 0.92±0.03 (n = 4), respectively. The moderate correlation in the two signal intensities in neutrophils, as compared to the other leukocyte types, was surprising. Therefore, the analysis was repeated in DFP-pretreated neutrophils (Fig. 3a) to inhibit serine proteases and apoptosis [24]. Under these conditions Pearson's coefficients increased to 0.78±0.2 (n = 8, p<0.05, Kolgomrov-Smirnov test). This result indicates that proteolytic degradation can spoil the detection of H_v_1 also in immunofluorescence experiments, resulting in the underestimation of H_v_1 and Nox2 colocalization. Among granulocytes PLB-985 cells displayed the most pronounced granular pattern, but in none of the tested leukocyte types localized H_v_1 dominantly to the cell surface. Weak or no signal was present over the nucleus in mature cells (the results obtained on mononuclear cells are available as Fig. S2a).

To verify the results obtained with confocal microscopy, WBs were performed on subcellular fractions of resting neutrophils. Confirming the results from confocal slices, significant H_v_1 signal could be detected in fractions of both granule (β_1_ and β_2_) and plasma membrane origin (γ, Fig. 3). The distribution of H_v_1 and Nox2 among the different fractions was similar, although not strictly correlated. Azurophil (α) granules, that are practically devoid of Nox2 [22], sometimes displayed marginal labeling for H_v_1 (not shown). In case of eosinophils, the strong colocalization with Nox2 suggests that H_v_1 localizes to the plasma membrane and to the membrane of small granules [23].

### Phagocytosis induces H_v_1 clustering

**Figure 4:**
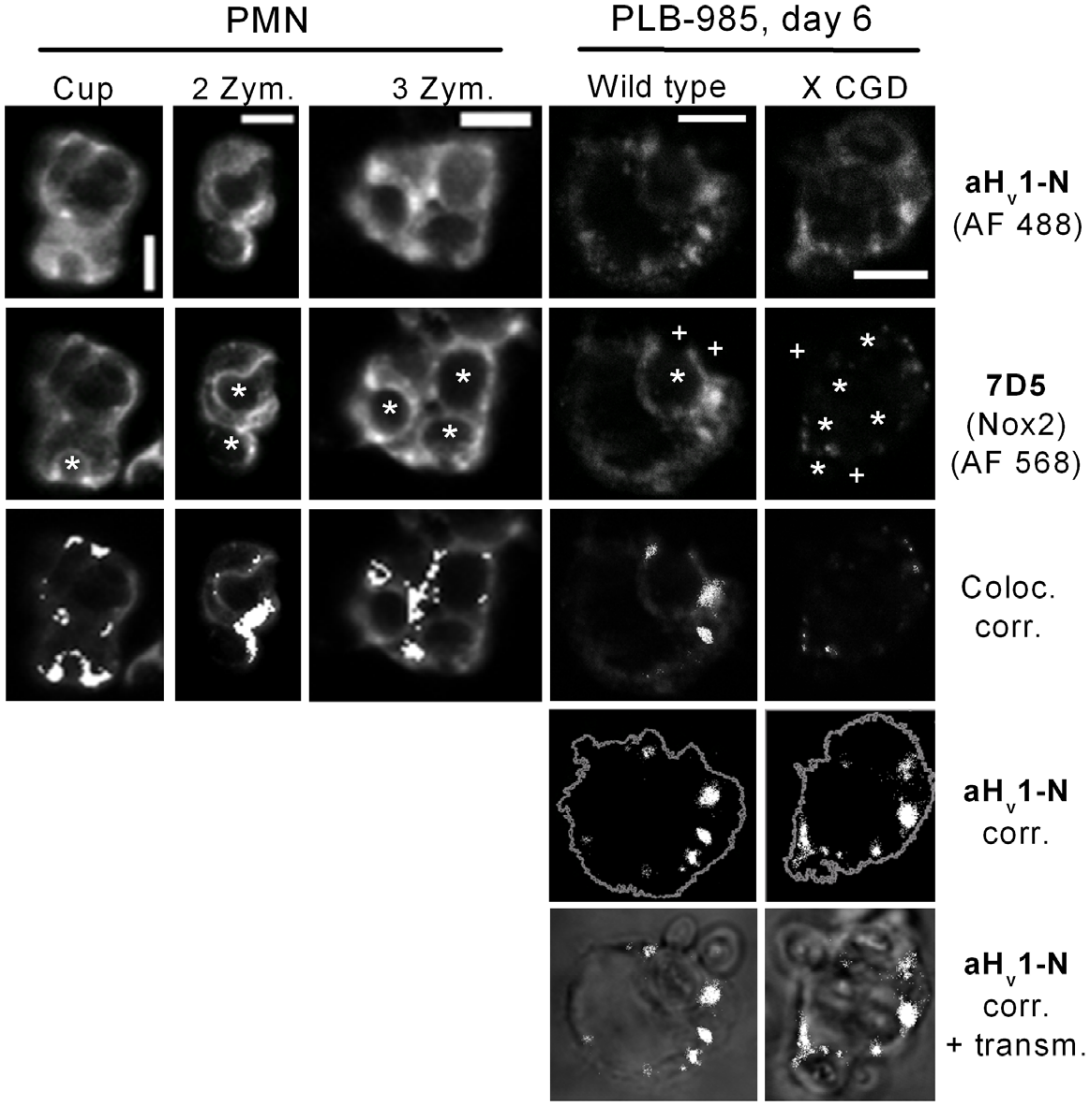
Partial intracellular colocalization of H_v_1 and Nox2 in phagocytosing granulocytes. H_v_1 (first raw) and Nox2 (second raw) tend to cluster during Zymosan phagocytosis, as detected in immunofluorescence experiments using confocal laser microscopy. The locations of zymosans are indicated by white asterisks. Forming (phagocytic cup, first column) and closed phagosomes (second and third column) are visible as round, hollow structures. In further analyses only pixels with intensity at least two times the average intensity (threshold) observed in experiments with control antibodies were included (activated phagocytes displayed significant, diffuse labeling in control experiments, not shown). Clusters of H_v_1 and Nox2 are often colocalized (third row). Colocalizing pixels are superimposed as white dots on the dimmed picture of 7D5 labeling (scale bars represent 5 µm). H_v_1 clustering is independent of Nox2 in PLB-985 cells (fourth raw). Cell perimeter (as derived form background labeling with H_v_1) is outlined in gray, and above-threshold H_v_1 labeling is presented as white dots. In the fifth raw above-threshold H_v_1 signals are superimposed as white dots over the (dimmed) visible light transmission image of phagocytosing cells. Pseudo color, 3D reconstructions of the cells in the 3rd and 4th columns are provided as supplemental Videos S1 and S2, respectively.

The prevailing view about the role of proton channel in phagocytes is that the channel is essential for the sustained activity of the phox during respiratory burst, mainly to provide compensatory charge for the depolarizing electron flow [9]. For such a task, proton channels should locate to the membrane compartment of intense electron transport, i.e. to the site of oxidase assembly and activity. Phagocyte NADPH oxidase promotes the antimicrobial activity of the phagosome during pathogen elimination [25], and assembly of phox subunits at the site of phagocytosis have been demonstrated [26], [27]. To explore whether H_v_1 is present on phagosomes, phagocytosis was induced by the addition of serum opsonized zymosan to purified, adherent granulocytes (more than 90% neutrophils) and to PLB-985 cells differentiated into granulcyte like cells. After 10 min the formation of phagocytic cups and closed phagosomes could be detected. Intense 7D5 labeling around engulfed zymosan particles indicated the accumulation of Nox2 in the phagosomal membrane (Fig. 4). Often overlapping with the 7D5 signal, the accumulation of aH_v_1-N labeling in zymosan surrounding areas was also pronounced in both cell types. The phagosomal accumulation of H_v_1 does not appear to require a functional oxidase, because strong clustering of the aH_v_1-N signal could also be detected in differentiated PLB-985 X CGD cells, a PLB-985 clone in which Nox2 is absent (i.e. the mutant, ∼56 kDa Nox2 gene product is not functional and is readily degraded) [28].

### H_v_1 expression and proton current density are correlated in PLB-985 cells

**Figure 5:**
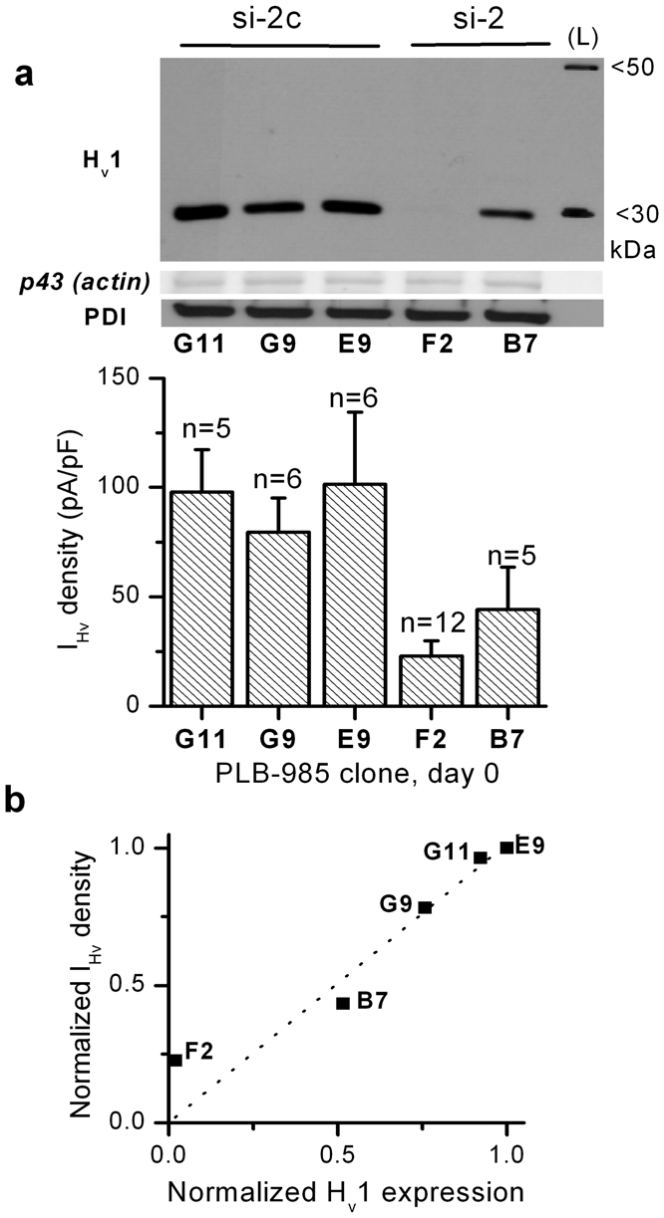
Correlation between H_v_1 and voltage-gated proton current. (**a**) The expression level of H_v_1 (top) and the corresponding I_Hv_ density (below) is presented in selected PLB-985 clones. The clones were transfected with a plasmid producing siRNA capable of knocking down H_v_1 expression (si-2) or its control siRNA (si-2c). For WB total cell lysates of 10^6^ cells were loaded each lane. The I_Hv_ density in clone F2 is significantly smaller than in E9 and G11 (p<0.05, Kruskal-Wallis test) (**b**) Mean I_Hv_ density value is plotted against the corresponding H_v_1 signal normalized to p43 signal, as measured with densitometry. The values of each clone are divided by the values of clone E9. Dotted line is the result of linear fit constrained to path through the origin (R>0.97, p<0.005).

Leukocytes from H_v_1 knockout mice lack voltage-gated proton currents, indicating that H_v_1 is essential for these currents in the mouse [12]–[14]. A similar genetical model is not available in human, as no inherited H_v_1 deficiency has been reported. Therefore, an ultimate proof is lacking to qualify H_v_1 as the proton channel molecule of human leukocytes. To investigate the putative proton channel role of H_v_1 in human leukocytes, three small interfering RNAs (siRNAs) were designed corresponding to sequences only present in the H_v_1 mRNA. Of the three siRNAs two (si-1 and si-2) efficiently knocked down H_v_1 expression in transfected COS-7 cells (not shown), as compared to their minimally altered control sequences (si-1c and si-2c). Of the two effective constructs only the si-2/-2c pair proved to be non-toxic in long-term cell culture experiments. To establish the relationship between H_v_1 level and proton current density in a human myeloid leukemia cell line, PLB-985 cells were stably transfected with plasmids containing puromycin resistance gene and a short hairpin RNA (shRNA) sequence encoding either si-2 or si-2c. After the analysis of H_v_1 protein expression in puromycin-resistant PLB-985 clones, three control and two H_v_1 knock-down clones were tested in whole-cell patch-clamp experiments (Fig. 5a). The results indicate that the density of characteristic voltage-gated proton currents and the H_v_1 expression level is well correlated in non-differentiated PLB-985 clones (Fig. 5b). Furthermore, the expression of the same shRNAs in a lymphoid leukemia cell line (Jurkat) also resulted in the reduction of proton current density (Fig. S3). These data support the view that H_v_1 is indispensable for voltage-gated proton currents in human leukocytes.

### H_v_1 and Nox2 expression is upregulated in parallel during granulocytic differentiation

**Figure 6:**
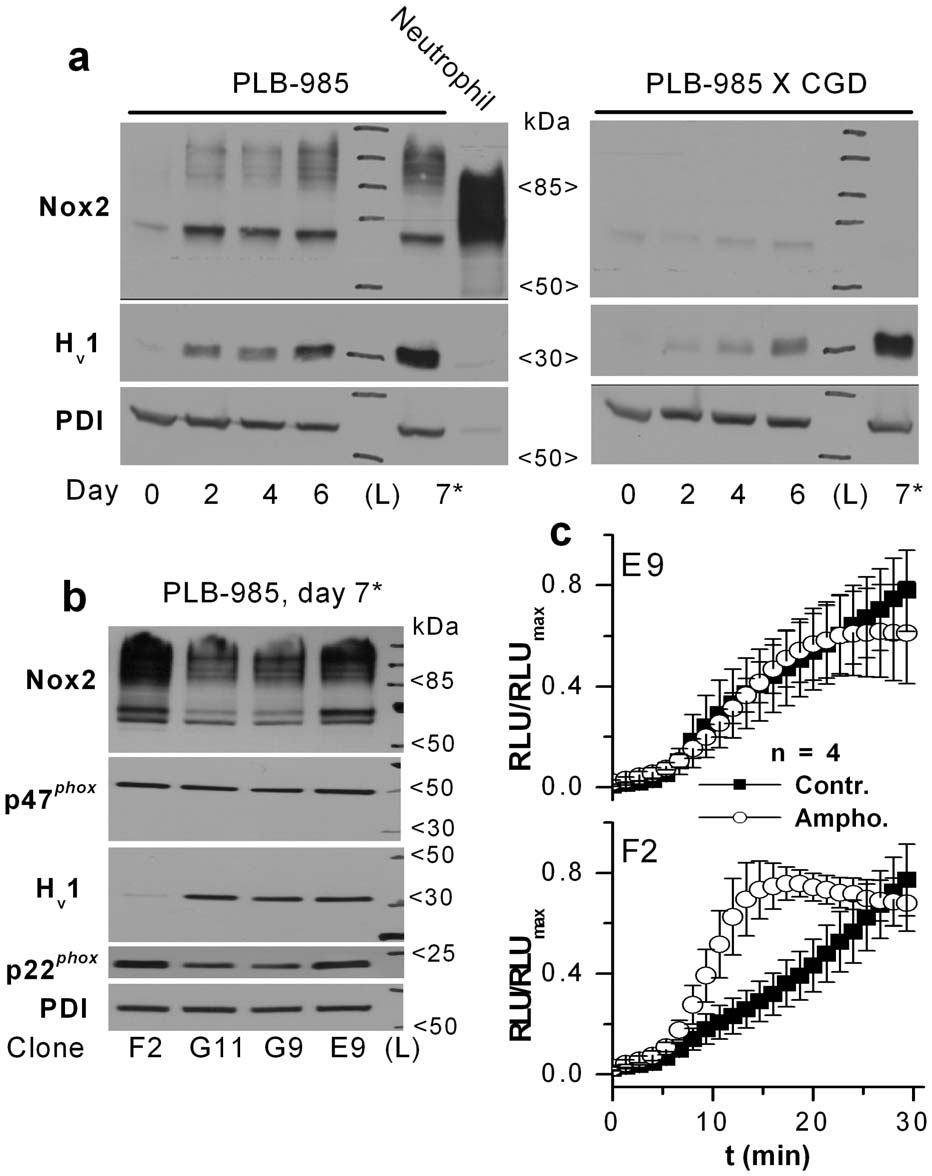
The functionally coupled H_v_1 and Nox2 are induced in parallel, but largely independently during granulocytic differentiation in PLB-985 cells. (**a**) Normal H_v_1 expression can be induced in the absence of normal Nox2 level. For WB total cell lysates of 10^6^ cells were loaded each lane. Samples of PLB-985 cells were prepared before (0) and 2, 4, 6 days after inducing differentiation with 0.5% DMFA. In a separate sample (7*) cells were differentiated for 7 days, and DMFA treatment was applied in low-serum culture medium (0.5% v/v) to increase the differentiation pressure. Different anti-Nox2 labeled bands correspond to different glycozilation states of the 65 kDa Nox2 protein. (**b**) The normal expression of different phox subunits (Nox2, p22*^phox^* and p47*^phox^*) is not disturbed by strongly reduced H_v_1 expression in differentiated PLB-985 cells. (**c**) Amongst differentiated PLB-985 clones amphotericin B amplifies superoxide production (2.92±0.48 times at 15 min, p<0.05, Mann-Whitney U test) only in clone F2, in which H_v_1 expression is strongly diminished. No significant change in superoxide production was detected in E9, G9 and B7 clones in the presence of amphotericin B. Diogenes^™^ reagent was used to detect the extracellular release of superoxide. Cells were preincubated for 15 min in a 1∶1 mixture of Diogenes^™^ and H-medium with or without 10 µg/ml amphotericin B. At time point 0 cells were activated by 200 nM PMA. Negligible Diogenes^™^ luminescence could be detected in PMA-treated, non-differentiated PLB-985 clones and during the preincubation period (not shown).

During granulocytic differentiation of human HL-60 leukemia cells the occurrence and development of proton conductance is paralleled by the functional and biochemical appearance of the phox [29]. We were interested whether the expression of the two proteins is by any means correlated during granulocytic differentiation. In PLB-985 cells, a subclone of HL-60 cells [30], the level of phox components also increases upon granulocytic differentiation [28]. We decided to use PLB-985 cells to study the relationship between H_v_1 and Nox2 expression, since a Nox2 gene-disrupted clone is also available (PLB-985 X CGD [28]). The expression level of both H_v_1 and Nox2 gradually increased after inducing differentiation with DMFA (Fig. 6a). The change in the amount of both proteins was most pronounced during the first two days. To test whether this parallel change in protein level reflects an interdependence of H_v_1 and Nox2 expression, the experiment was repeated in Nox2 deficient (X CGD) PLB-985 cells. Each tested day after starting the DMFA-induced differentiation the H_v_1 expression appeared reduced in Nox2 deficient cells (Fig. 6a). The difference in aH_v_1-N labeling of the two cell lines after 6-day long DMFA induction was also detected in immunofluorescence experiments (not shown). H_v_1 expression might be impaired in PLB-985 X CGD cells for the following reasons: a) PLB-985 X CGD clone has a general differentiation problem, b) PLB-985 X CGD is a clone in which H_v_1 expression is diminished in a Nox2 independent manner or c) normal Nox2 expression is required for normal H_v_1 expression. The latter does not seem to be a key factor, because no major difference in the H_v_1 level could be observed on day 7, if the differentiation pressure was increased by reducing the serum content of the culture medium (from 10 to 0.5% v/v [31], Fig. 6a).

The fact that the lack of functional phox does not have a dramatic impact on H_v_1 expression does not rule out that normal expression of phox subunits requires normal H_v_1 levels. To test the latter possibility, PLB-985 cell clones that express normal or diminished amounts of H_v_1 were differentiated for 7 days in DMFA containing, low-serum medium. As Fig. 6b demonstrates, massive reduction in H_v_1 level does not prevent high-level expression of the phox subunits Nox2, p22*^phox^* and p47*^phox^*.

### Reduced expression of H_v_1 can limit superoxide production

The rate of ROS production is significantly reduced in leukocytes from H_v_1 knockout mice [12]–[14]. Supposing that the role of H_v_1 is the same in mouse and human leukocytes, one should observe reduced ROS production in cells that display decreased H_v_1 expression. Surprisingly, however, in pilot experiments the PMA-induced superoxide production of the H_v_1 knock-down F2 and B7 PLB-985 clones exceeded that of G9 and G11 control clones, and was only inferior to that of clone E9 (not shown). We suspected that this result might be explained by the large heterogeneity in the Nox2 expression level of PLB-985 clones (Fig. 6b). The PMA-induced ROS production of H_v_1^-/-^ mouse granulocytes was reported to be normalized by incorporating monovalent cation channels (gramicidin peptides [32]) into their cell membrane [14]. Although the conductances for the monovalent cations through gramicidin channels are ranked in the same order as their aqueous mobilities (i.e. proton moves fastest [32]), it is unlikely that significant proton extrusion through gramicidin was possible in the presence of normal intracellular K^+^ concentration. This indicates that charge compensation, rather than proton removal, became the bottleneck for ROS production in PMA-activated H_v_1 deficient cells. If the superoxide production of a differentiated PLB-985 clone in which H_v_1 expression is knocked-down is also limited by shortage of compensatory charge flow, we should be able to compensate for it using an artificial conductance. Therefore, we measured the PMA-induced extracellular superoxide release of two control (E9, G9) and two H_v_1 knock-down (F2, B7) clones with or without preincubation with the ion channel forming antibiotic amphotericin B. In the presence of amphotericin B only F2 clone displayed significant increase in PMA-induced superoxide production, and only in its early phase (Fig. 6c). Superoxide production declined after ∼20 min in the presence of Amphotericin B, an effect which was not specific for clone F2. As clone F2 express high level of Nox2 but very few H_v_1, the above results support the notion that charge movement through H_v_1 can become rate-limiting for very intense ROS production in human cells.

## Discussion

A decade-long debate about the identity of the voltage-gated proton channel molecule came to an end when H_v_1 (from human [3] and mouse [2]) was cloned in 2006. Many publications since have investigated the expression, structure and possible role of H_v_1 in heterologous expression systems and in mouse leukocytes. On the other hand, data on H_v_1 in human leukocytes [20], especially in granulocytes, are scarce. This study was aimed to define the main characteristics of H_v_1 in human granulocytes.

### H_v_1 supports voltage-gated proton current in human leukocytes

It has been taken for granted that H_v_1 is the proton channel molecule in human leukocytes, but it has never been formally demonstrated. Now we have found that the expression levels of H_v_1 in major types of human blood leukocytes (Fig. 1) correspond well to proton current density data obtained in these cells. T-cells display only tiny proton currents (0.9 pA/pF [1]) and the expression of H_v_1 mRNA and protein in these cells, if any, is below the reliably detectable level. In B-cells and granulocytes the abundant expression of H_v_1 protein is in accordance with their pronounced proton current amplitudes (∼17–200 pA/pF [1]). Peripheral blood monocytes also display remarkable H_v_1 expression, but electrophysiological data is not available about voltage-gated proton currents in this cell type. Nevertheless, the presence of such currents in macrophages and in THP-1 monocyte like leukemia cell line has been extensively demonstrated [1]. In line with the above data, in our experiments knocking down H_v_1 expression in leukemia cell lines PLB-985 and Jurkat, resulted in reduced I_Hv_ density (Figs. 5 and S3, respectively). Taken together, the available data strongly support the notion that H_v_1 expression is essential for voltage-gated proton currents in human leukocytes.

### H_v_1 is capable of forming stable dimers in human granulocytes

The dimeric nature of H_v_1 had been extensively investigated and characterized in heterologous expression systems [6]–[8]. Available results indicate the role of the C- [7], [8], [19] and N-terminal [6], [8] intracellular domains and of the extracellular loop between S1-S2 transmembrane helices [7] in dimerization. Importantly, monomers and dimers of H_v_1 give rise to kinetically distinct I_Hv_s. Monomeric H_v_1 activates faster upon depolarization [8], while subunits in the dimer display cooperative behavior [33]. Based on the above results, it was suggested that the differences in I_Hv_ kinetics of different cell types and of resting and activated phagocytes might be explained by differences in the dimer to monomer ratio of H_v_1. Now we have demonstrated for the first time that in case of endogenously expressed H_v_1, the dimer form is also abundantly present in all major types of human leukocytes and in differentiated PLB-985 cells (Figs. 2 and S2b). The existence of distant dimerization sites (see above) could theoretically allow the formation of higher-order H_v_1 multimers (e.g. tri- or tetramers) as well, supposing that H_v_1 density is high enough in the membrane. However, in spite of various attempts, we could not prove significant formation of such multimers in human granulocytes.

The H_v_1 dimer is stabilized, at least in part, by strong polar interactions in granulocytes, because high concentration of ionic detergent (4% SDS) was required to efficiently disrupt them (Fig. 2a). On the other hand, disulfide formation between monomers appears to be a minor contributor of dimer stabilization. The human H_v_1 contains only two cysteines, of which cys249 in the C-terminal intracellular domain is positioned so that it is amenable to dimer stabilization [7], [19]. Similar to the data obtained with heterologously expressed H_v_1 [7], [19], our results with SH-reagents indicate that this cysteine is in reduced form also in resting granulocytes (Fig. 2b). Redox compounds, e.g. cytoplasmic NADPH [15] or ROS [18], could theoretically affect dimer stability through cys249, but further experiments are required to prove that such changes actually occur and influence the properties of I_Hv_. Nevertheless, our results with SH-reagents do not indicate a major change in the redox state of this cysteine upon PMA-induced respiratory burst (Fig. 2b). The C-terminal domain of H_v_1 forms coiled-coil [19], a structure often involved in interactions between ion channel subunits, and it seems to be involved in H_v_1 dimer stabilization [7], [8], [19]. The role of the largely unstructured N-terminal intarcellular domain [7] in dimer formation is less clear. The artificial removal of its intracellular C-terminal domain led to diminished dimer formation by H_v_1, a phenomenon that was exacerbated by the concomitant removal of the N-terminal intracellular domain [8]. In our experiments H_v_1 appeared prone to cleavage by proteases (Figs. 1c and 3a). How cleavage of intracellular domains could contribute to the changes in I_Hv_ phenotype upon granulocyte activation is not clear, since those changes are largely reversible [15], [34]. Nevertheless, the cleavage and/or degradation of the N-terminal domain may still possess a more general regulatory potential in leukocytes, as its overexpression inhibited the proliferation of a premature B-cell lymphoma cell line [35]. In summary, our data do not support the notion that granulocyte activation induces changes in dimer to monomer ratio, as the efficiency of crosslinking was independent of PMA addition (Fig. 2b). Furthermore, based on indirect evidences, the idea that monomer-dimer interconversion occurs during activation of phagocytes was recently challenged by others as well [36].

### H_v_1 resides mainly in granular membranes in human granulocytes

The heterologously expressed H_v_1 was confined to intracellular membranes in HeLa cells in a C-terminus dependent manner. On the other hand, native H_v_1 was mainly detected in the plasma membrane and in endosomes of human B-cells [20] (see also Fig. S2a). Our results obtained with subcellular fractionation and confocal imaging concordantly demonstrate that in case of resting human granulocytes H_v_1 is mainly located to the membrane of intracellular granules, but its presence at the plasma membrane is also significant (Figs. 3b–c).

### The functionally coupled H_v_1 and Nox2 are expressed largely independently

Voltage-gated proton channel is viewed as a well-suited tool to support the intense transmembrane electron transport by the phox during phagocyte respiratory burst [11]. In line with this view, activation of the phox promotes proton channel activity by speeding up its activation, delaying its deactivation and shifting its threshold potential to more negative values [1], [37]. Additionally, recent reports demonstrated significant reduction in the ROS producing capability of PMA-activated neutrophils [12], [14], bone marrow cells [13] and B-cells [20] from H_v_1 KO mice. Notably, these cells were devoid of I_Hv_. This strong functional coupling between H^+^ and e^−^ transport prompted us to investigate, how the expression and distribution of H_v_1 and Nox2 are related in human leukocytes. The strong functional coupling of H_v_1 and phox is nicely reflected in their expression, because a) Nox2 and H_v_1 display colocalization in different types of phagocytes (Figs. 3a and S2a), b) their expression is induced in parallel during granulocytic differentiation in PLB-985 cells (Fig. 6a) and c) both proteins tend to accumulate in the phagosome membrane during zymosan engulfment (Fig. 4). It has to be emphasized, however, that the accumulation of H_v_1 in phagosomes and the early and massive induction of H_v_1 expression during differentiation occur in PLB-985 cells even if functional Nox2 is absent (Figs. 4 and 6a). In analogy with the latter result, resting granulocytes from Nox2 deficient patients display normal I_Hv_ density [1]. Furthermore, we could not detect Nox2 labeling in many of the lymphocytes, which labeled strongly for H_v_1 (Fig. S2a). The above data confirm that high level H_v_1 expression does not require the presence of Nox2. Conversely, a massive reduction in H_v_1 expression does not interfere with the intense expression of phox subunits in PLB-985 cells (Fig. 6b). In this respect it is important that leukocytes from H_v_1 KO mice express the phox subunits rather normally [12], and exhibit normal Nox2 mediated electron currents [14], [38]. Although H_v_1 and phox are practically independently expressed, the available data indicate that H_v_1 deficiency leads to restricted phox activity in mouse leukocytes [12]–[14]. Importantly, no naturally occurring human H_v_1 deficiency has been reported to date. Chances, however, seem to be low for finding such a case, because, unlike in the mouse, high level H_v_1 expression is likely required for normal fertility in human [39]. Our results obtained using RNA-interference now demonstrate that severely reduced H_v_1 expression can become a limiting factor for superoxide production also in granulocytes differentiated from the human leukemia cell line PLB-985 (Fig. 6c).

### Is H_v_1 the only proton channel in neutrophils?

Several lines of evidence indicate that H_v_1 functions as a proton channel in mouse and human leukocytes. The fact that H_v_1 is a proton channel does not rule out that another proton channel may exist and might be involved in supporting ROS formation in granulocytes. For many years Nox2 was viewed as a potential candidate to mediate proton current in human granulocytes, especially upon activation of ROS production [16], [17]. In this respect it is interesting to note that H_v_1 deficient mouse leukocytes display significant ROS production [12]–[14]. Similarly, our PLB-985 clones are capable of intense ROS production even if H_v_1 expression is severely damaged. These findings may give the impression that H_v_1 is not the only protein that can attenuate the charge separation by Nox2. Therefore, one has to pose the following questions. Is another proton channel present in neutrophils? If no other proton channel is present, how can one explain the above contradictions? The strongest evidence against an ancillary proton channel was obtained in H_v_1 deficient mouse neutrophils. These cells display normal electron currents upon activation, but do not show any trace of voltage-gated proton current under resting and PMA-activated conditions [14], [38]. This indicates that these cells assemble functional oxidase at the plasma membrane, but neither Nox2 nor another channel seem to conduct protons, at least not in a depolarization-activated fashion. Whether Nox2 or another molecule is capable of conducting protons in a voltage independent manner remains to be tested. How can one explain the significant ROS production in H_v_1 deficient mouse leukocytes? It is very likely that in a KO system other transporters can partially compensate for the absence of voltage-gated proton channel activity. Furthermore, potassium and chloride channels had been suggested to participate in charge compensation even in normal granulocytes [25]. Whether such mechanisms are upregulated in the absence of H_v_1 remains to be elucidated. Although some controversy exists [1], [16], [17], it is likely that similar to the mouse ortholog human Nox2 does not mediate voltage-gated proton current. In line with this assumption, the density of voltage-gated proton current correlates well with H_v_1 (see above), but not with Nox2 [1] expression among different human leukocyte types and among PLB-985 clones (Fig. 5).

In summary, beside monomers endogenously expressed H_v_1 forms stable proton channel dimers in resting and activated human granulocytes. The characteristics and expression pattern of H_v_1 is optimized to support intense phox activity. As the role of ROS production may not be the same in all leukocytes [20], [40], the largely independent expression of H_v_1 and phox components allows fine-tuning of their teamwork.

## Materials and Methods

### Solutions

For patch-clamp recordings the bath solutions contained (mM): CsCl 1, tetraethylammonium chloride 1, MgCl_2_ 2, EGTA 1, N-methyl-D-glucamine base 101 and HEPES acid 200 (pH 7.55). The pipette solution contained in (mM): CsCl 1, tetraethylammonium chloride 1, MgCl_2_ 2, EGTA 1, N-methyl-D-glucamine base 101 and MES acid 200 (pH 6.15). H-medium contained (mM): NaCl 145, KCl 5, MgCl_2_ 1, CaCl_2_ 0.8, HEPES 10 and glucose 5 (pH adjusted to 7.4 with NaOH). Disuccinimidyl suberate (DSS), N-ethylmaleimide (NEM), *N*,*N*′-(1,3-phenylene)dimaleimide (PDM) were dissolved in DMSO at 250 mM, 400 mM and 10 mM, respectively. Amphotericin B and phorbol-12-myristate-13-acetate (PMA) were first dissolved in DMSO at 80 mg/ml and 5 mM, respectively, then diluted in H-medium to yield stock concentrations of 1 mg/ml and 20 µM, respectively. Phenylmethanesulfonyl fluoride (PMSF) was dissolved in ethanol at 100 mM.

### Cell culture and transfection

All cell lines but PLB-985 X CGD (see below) were purchased from ATCC-LGC (www.lgcstandards-atcc.org) and cultured following the instructions provided by ATCC-LGC with minor modifications, as follow. COS-7 cells were cultured in Dulbecco's modified Eagle's medium (GibcoBRL, Csertex kft, Hungary) supplemented with 10% heat inactivated fetal calf serum (FCS, GIBCO, Invitrogen, www.invitrogen.com), 100 i.u./ml penicillin and 100 µg/ml streptomycin. PLB-985 and Jurkat cells were grown in suspension in complete RPMI 1640 medium supplemented with 10% FCS, 100 i.u./ml penicillin and 100 µg/ml streptomycin. For transfection COS-7 cells were plated in 30 mm plastic tissue culture dishes (Greiner Bio-One, www.greinerbioone.com) and transfected (1 µg plasmid DNA) one day after plating using Lipofectamine 2000 (Invitrogen, www.invitrogen.com). Leukemic (PLB-985 and Jurkat) cells were transfected by electroporation (Amaxa Nucleofector Device, Amaxa Biosystems, www.amaxa.com), using the Cell Line Nucleofector Kit V from (Amaxa Biosystems). For establishing clones from puromycin (1 µg/ml) resistant cell populations the limiting dilution method was used. For differentiation of PLB-985 and PLB-985 X CGD ([28] generously provided by Mary C. Dinauer, Indianapolis) cells into neutrophil granulocyte like cells, the culture medium was supplemented with 0.5% v/v dimethylformamide (DMFA) with or without reducing the medium's FCS content to 0.5% v/v (to promote differentiation [31]).

### Isolation of blood cells and granule fractions

Blood cells were prepared from venous blood drawn from healthy adults after obtaining their informed and written consent. Red blood cells (RBCs) and leukocytes (WBCs) were separated by dextrane sedimentation. To separate mononuclear cells (MCs) from granulocytes (PMNs), WBCs after dextran sedimentation were layered on Ficoll-Paque Plus (GE Healthcare, www.gelifesciences.com) and centrifuged at 400 g for 20 minutes. Remaining RBCs were hemolysed by 30 s exposure to distilled water, followed by reconstitution of the osmolality with equal volume of 1.8% w/v NaCl in distilled water and by centrifugation (400 g, 5 minutes). Pellets containing PMNs or MCs were used to further separate fractions of WBCs by postive or negative selection utilizing fluorochrome or paramagnetically labeled antibodies (MicroBeads) and magnetic separator (VarioMacs) purchased from Miltenyi Biotec (www.miltenyibiotec.com). Eosinophil (CD16-) and Neutrophil (CD16+) fractions of PMNs were separated using CD16 MicroBeads. Monocytes were purified from MCs through positives selection with CD14 MicroBeads. After monocyte depletion of MCs, the remaining lymphocytes could be further dissociated into T-cells and B-cells on a FACSvantage DIVA cell sorter (Becton Dickinson, San Jose, CA) using CD3- or CD19-specific antibodies, respectively (anti-CD3-PE mAb, Beckman Coulter, Fullerton, CA; anti-CD19-FITC mAb, BD Pharmingen, San Diego, CA).

Garnule fractions of human neutrophils were prepared and tested for their identity and purity as described in Ref. [41]. Only preparations that provided the expected antigenicity profile were used. Membrane fractions were boiled for 5 min in reducing Laemmli sample buffer and stored at -70 C until use. The following fractions were separated: azurophil granule (α), specific granule (β_1_), gelatinase granule (β_2_) and secretory vesicle along with plasma membrane (γ).

### Western blotting

Sample buffers contained 5% v/v β-mercaptoethanol as reducing agent unless otherwise specified. Samples were not heat treated unless otherwise stated. Mononuclear leukocytes were lysed on ice in 2x Laemmli sample buffer. Unless otherwise specified, for lysing granulocytes and PLB cells, 4x Laemmli sample buffer was mixed with equal volume of distilled water (dH_2_O) supplemented with 5 mM EDTA (250 mM stock in dH_2_O, pH 7.4 with NaOH) and protease inhibitor cocktail (1 Complete Mini tablet in 10 ml dH_2_O, Roche Applied Science, www.roche-applied-science.com). When indicated, granulocytes and PLB-985 cells were incubated in nominally calcium and magnesium free media (RPMI 1640 with 5 mM EDTA or PBS) supplemented with diisopropylfluorophosphate (DFP, 1∶5000) for 30 min on ice before lysis. DSS crosslinking was performed for 20 min on room temperature. The reaction was quenched by 20 mM TRIS-HCl (pH 8.0). Reactions with NEM (20 mM), PDM (50 µM) or PDM + DSS were carried out on ice for 40 min on ice. PDM crosslinking was quenched with 20 mM NEM. These reagents are lipophil, and were added directly to cells (10^6^ cells in 1 ml sterile PBS) in the presence of DFP. After stopping the reactions, cells were pelleted by centrifugation (at 200 g for 3 min) and lysed in 30 µl 2x Laemmli sample buffer supplemented with 2 mM PMSF. Samples were run on 8 or 10% polyacrylamid gel and blotted onto nitrocellulose membrane. To block non-specific binding sites in Western blot experiments (WB), 5% w/v skimmed milk powder was applied in phosphate buffered saline (PBS, pH 7.4) for 1 hour. After incubating the membranes with the first antibody (rabbit polyclonal or mouse monoclonal) for 1 hour, membranes were washed 5 times in PBS 0.1% v/v Tween20. Horseradish peroxidase labeled anti-rabbit or anti-mouse secondary antibody was added in 1∶5000 dilution (in PBS, 0.1% v/v Tween20, 1% w/v skimmed milk powder) for 40 min, followed by washing five times in PBS 0.1% v/v Tween20. Signals were detected on FUJI Super RX films (Fujifilm, www.fujifilm.com) using the enhanced chemiluminescence method (GE Healthcare, ECL™ Western Blotting Analysis System).

### Immunofluorescent labeling

In immunofluorescence experiments (IF) cells attached to coverslips were fixed in 4% w/v paraformaldehyde in phosphate buffered saline (PBS, pH 7.4) then rinsed 5 times in PBS and incubated for 10 minutes in PBS containing 100 mM glycine. Coverslips were washed 2 times in PBS and cell permeabilization was carried out in PBS containing 1% w/v bovine serum albumine (BSA) and 0.1% v/v Triton X-100 for 20 min. To block non-specific binding sites (e.g. Fc-receptors of WBCs) 10% v/v pooled human serum (from at least 3 healthy donors, self made), 10% v/v normal goat serum, 10% v/v human Fc-receptor blocking reagent (Miltenyi Biotec) and 1% w/v bovine serum albumin were applied in PBS for 1 h. Coverslips were then incubated with the primary antibody, washed thoroughly 6 times in PBS and incubated with the secondary antibody for 1 hour and finally washed 6 times in PBS again. Coverslips were mounted using Mowiol 4–88 antifade reagent (prepared from polyvinyl alcohol 4–88, glycerol, H_2_O and TRIS pH 8.5). In IF 5% v/v pooled human serum, 5% v/v normal goat serum, 5% v/v human Fc-receptor blocking reagent and 1% w/v bovine serum albumin were present during the application of antibodies.

### Antibodies

To detect human voltage-gated proton channel, affinity purified rabbit polyclonal antibody was used at 2 (WB) or 4–8 (IF) µg/ml. To produce polyclonal anti H_v_1- antibody (aH_v_1-N), female white rabbits were immunized with the N-terminal 99 amino acids of H_v_1 tagged with glutathion-S-transferase. The same peptide construct (covalently bound to Affi-Gel 15 media, Bio-Rad Laboratories, www.bio-rad.com) was used for affinity purification of aH_v_1-N, after the serum of the immunized rabbit was depleted for anti-glutathion-S-transferase antibodies. As negative control for aH_v_1-N normal rabbit Ig-G (Santa Cruz Biotechnology, www.sbct.com) was applied at 8 µg/ml. To detect gp91*^phox^* with immunofluorescence, supernatant of the mouse monoclonal hybridoma 7D5 [21] was used at 20-time dilution, and purified mouse IgG1 (BD Biosciences Pharmingen, www.bdbiosciences.com) at 2.5 µg/ml served as isotype control. Monoclonal anti-gp91*^phox^* antibody (m48ab [42]) was generously provided by Dr Dirk Roos. To detect p47*^phox^* or myeloperxoidase, Cell Signaling Technology #4312 or #4162 (www.cellsignal.com) antibody was used, respectively. Anti-p22*^phox^* antibody (SC-20781) was purchased from Santa Cruz Biotechnology Inc. (www.scbt.com). Antibodies against gelatinase and CD14 were from Abcam (ab76003 and ab45870, respectively, www.abcam.com). Anti-lactoferrin antibody (L3262) was purchased from Sigma-Aldrich (www.sigmaaldrich.com). For loading control in Western blots anti-protein disulphide isomerase antibody (aPDI) was used (ab2792, Abcam). Horseradish peroxidase labeled secondary antibodies were from GE Healthcare (for detection with GE Healthcare, ECL™ Western Blotting Analysis System). Alexa Fluor® 488- and Alexa Fluor® 568-labeled secondary antibodies were from Molecular Probes (probes.invitrogen.com).

### Confocal laser scanning microscopy and colocalization analysis

Confocal images were collected on an LSM 510 laser scanning confocal unit (Carl Zeiss, www.zeiss.com) with a 63X 1.4 numerical aperture plan Apochromat and a 40X 1.3 numerical aperture plan Neofluar objective (Carl Zeiss). Excitation was with 25 mW argon laser emitting 488 nm, and a 1.0 mW helium/neon laser emitting at 543 nm. Emissions were collected using a 500–530 nm band pass filter to collect Alexa Fluor® 488, and a 560 nm long pass filter to collect Alexa Fluor® 568 emission. Images from optical slices of 1 µm thickness were acquired. LSM software (Carl Zeiss) was used for image acquisition. For analyzing images the ImgaeJ software was applied (Rasband, W. S., U.S. National Institutes of Health, Bethesda, MD, http://rsb.info.nih.gov/ij/). For image processing ImageJ and Pain.Net 3.5 free image editing software (www.paint.net) were used. Analysis of H_v_1 and Nox2 colocalization was performed only in cells in which at least moderate labeling (two times above background) for both proteins could be detected. For quantification of colocalization the Pearson's coefficient was used [43]. Its value (r) can range from 1 to −1 with r = 1 standing for complete positive correlation and r = −1 for a negative correlation, with r = 0 standing for no correlation in pixel intensities of the two channels. To demonstrate the extent of colocalization in Figs., the colocalization highlighter plugin of the ImageJ software was used. Before applying the plugin, image pairs were corrected for differences in mean signal intensities between the two emission wavelengths. Additionally, pixels with below threshold intensity were excluded from analysis. Unless otherwise stated, threshold was set to 20% of maximal detectable signal intensity. Pixel pairs with intensity ratio between 0.6 and 0.6^−1^ are displayed as colocalizing.

### Phagocytosis experiments

Zymosan was opsonized with pooled human serum (from at least 3 healthy donors) by 30-min incubation at 37°C. PMNs (10^8^/ml) or 6-day long differentiated PLB-985 cells were allowed to adhere on fibronectin coated glass coverslip for 10 minutes in H-medium. To induce phagocytosis, zymosan was added to phagocytes for 10 min at 37°C to a final concentration of 300 µg/ml.

### Detection of superoxide release

To measure extracellular superoxide release, differentiated (for 6–7 days with DMFA) PLB-985 cells were suspended at ∼10^6^/ml in a 1∶1 mixture of Diogenes™ superoxide detection medium (National Diagnostics, www.nationaldiagnostics.com) and H-medium. Aliquots of the suspension (100 µl) were added into wells of white, 96-well plate and prewarmed at 37°C for 15 min. Superoxide induced chemiluminescence was detected at 37°C by a shaking fluoro-luminometer (Ascent Fluoroscan FL, Thermo Scientific, www.thermo.com).

### Molecular biology

For cloning of H_v_1 total RNA from mature dendritic cells was isolated with Trizol reagent (Invitrogen, www.invitrogen.com). cDNA was synthesized from 2.5 µg total RNA using oligo(dt)_18_ primers and RevertAid M-MuLV Reverse Transcriptase (Fermentas, www.fermentas.com) in 20 µl reaction mix. The open reading frame of H_v_1 was TA cloned into pCDNA3. 1/V5-His-TOPO vector (Invitrogen) with High Fidelity PCR Enzyme (Fermentas) using 1 µl of the first strand as template. The sequence was confirmed by sequencing reactions (Eurofins MWG Operon, www.eurofinsdna.com). For real-time RT-PCR (qPCR) total RNA was isolated as above then treated with DNaseI (Ambion, Austin, TX). Reverse transcription was performed from 100 ng total RNA using Superscript II reverse transcriptase (Invitrogen) and random hexamers (Applied BioSystems, Warrington, United Kingdom) using a standard RT reaction. Amplification reactions were performed in an ABI PRISM 7900 sequence detector (Applied Biosystems) using 40 cycles of 94°C for 12 seconds and 60°C for 1 min. All PCR reactions were done in duplicates with one control reaction not containing RT enzyme. The comparative Ct method was used to quantify the amount of HVCN1 relative to cyclophilin. The following PCR primers and probes were used (5′–3′): forward primer for cyclophilin (NM_021130) ACGGCGAGCCCTTGG, reverse primer TTTCTGCTGTCTTTGGGACCT. Probe was FAM-CGCGTCTCCTTTGAGCTGTTTGCA-BHQ_1. Taqman gene expression assay for HVCN1 was purchased from Applied Biosystems (Assay ID: Hs00260697_m1). Sequences coding small hairpin RNAs designed to knock down H_v_1 (HVCN1, transcript variant 1) mRNA level and their minimally changed control sequences were cloned into one of STRIKE™ U6 plasmid vectors (Promega, www.promega.com) either containing green fluorescent protein (Cat.# C3550) or puromycin resistance gene (Cat.# C7900) as selection marker. The following target sequences were used (5′–3′): GAACGGCAACTCTTAAGGT (si-1), GAACCGGAACTCTTAAGGT (si-1c), GGTGGCCCGGATCATCAAT (si-2), GGTGGCGCGCATCATCAAT (si-2c).

### Patch-clamp measurements

Whole-cell voltage-clamp recordings were performed with an Axoptach-1D patch-clamp amplifier (Axon Instruments, Foster City, CA) equipped with a CV-4-1/100U headstage. Pipettes were pulled from borosilicate glass tubing (type 1B120F-4, World Precision Instruments, Inc., Sarasota, FL) using a P-87 puller (Sutter Instrument Co., CA). After fire polishing, the pipette resistance was 7–13 MΩ when filled with the recording solution. The bath was grounded using an Ag/AgCl pellet. Current signal was low-pass filtered at 100–200 Hz (−3 dB, 8-pole Bessel filter) and sampled at 250–500 Hz. Data acquisition and analysis were performed using pClamp 6 and 8 software (Axon), respectively. To ensure that voltage-gated proton currents were analyzed, reversal potential of the depolarization-activated current was defined using ramp-tail currents, as demonstrated in Fig. S3 and described earlier in detail [15].

### Data analysis

Data are presented as mean ± S.E. unless otherwise stated. Statistical analyses were performed using the Statistica software (version 8, Statsoft, Inc., Tulsa, OK, USA). WB and IF experiments were performed at least twice independently unless otherwise stated in the figure legends.

When using transfection reagents, cell labeling kits or other special material the instructions of the manufacturer were rigorously followed. Chemicals were obtained from Sigma-Aldrich unless otherwise specified. All manipulations were performed at room temperature (23–28°C) unless otherwise stated. The studies conformed to the standards set by the Declaration of Helsinki, and the procedures were approved by the ethics committee of Semmelweis University and University of Debrecen.

## Supporting Information

Figure S1 A polyclonal antibody selectively recognizes native Hv1. To test the specificity and sensitivity of the antibody, COS-7 cells were transiently transfected with plasmid encoding V5-epitope tagged full length Hv1 (Hv1-V5). (a) Detection of colocalization of anti-V5 and aHv1-N labeling in Hv1-V5 transfected COS-7 cells in immunofluorescence experiments using confocal laser scanning microscopy (Pearson's coefficient is 0.87). The two antibodies label the same cells and cellular structures. Secondary antibodies were labeled with Alexa Fluor® (AF, scale bar, 50 µM). In mock transfected cells only very weak auto fluorescence was detectable (not shown). (b) Result of colocalization analysis performed on pixels with above threshold intensities (see methods). Colocalizing pixels are displayed as white dots in the most right column. (c) Anti-V5 and aHv1-N both detect Hv1-V5 in COS-7 cells transfected with the construct (C+), but not in mock transfected (C-) cells. Only aHv1-N recognizes native Hv1 in human eosinophils (Eo.). Labeling the house keeping enzyme PDI with aPDI served as loading control. The estimated molecular weights (m.w.) of the Hv1 monomer and our Hv1-V5 construct are ∼31.6 and ∼36.5 kDa, respectively.
(4.81 MB TIF)

Figure S2 Localization and structure of Hv1 in human leukocytes. (a) Detection of Hv1 (most left column) and Nox2 (middle) in mononuclear cells. Colocalization analysis was performed only in cells in that at least moderate labeling (two times above background) for both proteins could be detected. Above threshold colocalizing pixels are displayed as white dots in the most right column. After correction for differences in mean signal intensities between Hv1 and Nox2 labeling, threshold was set to 20% of maximal detectable intensity. Monocytes displayed strong labeling with aHv1-N, sometimes with strong, colocalizing 7D5 signal. Based on their staining pattern, lymphocytes could be classified into two major groups. Cells in the first group were not stained with either of the two antibodies (not shown), while those in the other group showed strong aHv1-N labeling with strong (lymphocyte 1) to background (lymphocyte 2) 7D5 signal. In lymphocytes aHv1-N labeling often displayed a patchy pattern, which likely arises from endocytotic vesicles (as demonstrated earlier by others [Capasso M. et al., 2010, Nat Immunol 11: 265–272]). Pearson's coefficient for lymphocytes is 0.80±0.02 (n = 4). Scale bars represent 5 µm; Pearson's coefficient for the displayed monocyte is 0.92. (b) Demonstration of Hv1 dimers in human mononuclear leukocytes after cross-liking with DSS in WB after a standard reducing PAGE. Cells were preincubated with DFP (1∶5000), and DFP was present during crosslinking.
(3.80 MB TIF)

Figure S3 Hv1 supports voltage-gated proton current in the human lymphoid leukemia cell line Jurkat. (a) Representative curves from whole cell patch-clamp recordings demonstrate voltage-gated proton current in Jurkat cells transfected with control siRNA, si-2c. Every 15 s, a long-lasting (3 s, A) or short-lasting (0.2 s, B) activating pulse to 80 mV was applied, followed by a rapid voltage ramp to the -90-mV holding potential (C, D). The reversal potential of the voltage- and time-dependent current (inset) was determined by subtracting the two “ramp tail” currents (C–D). (b) Representative curves demonstrate voltage-gated proton current in Jurkat cells transfected with a siRNA (si-2) effective in knocking down Hv1 protein in Hv1-V5 transfected COS-7 cells (not shown). Recordings of transiently transfected Jurkat cells were performed 48–96 h after transfection. Transiently transfected Jurkat cells were identified based on their GFP fluorescence by illuminating the cells at 488±12 nm using a monochromator equipped 75 watt xenon arc lamp of a monochromator equipped fluorimeter (PTI, South Brunswick, NJ, USA). (c) Voltage protocols used in a and b. (d) Cumulative data of proton current density (amplitude of the time dependent current component normalized to whole cell capacitance) in Jurkat cells transfected transiently with control (si-2c) or effective (si-1 and si-2) siRNA constructs. Si-1-1-C1 is a puromycin selected clone stably expressing si-1. (e) Western blot detection of Hv1 in Jurkat cells. Expression level of the ∼36 kDa apparent m.w. protein is reduced in puromycin selected Jurkat cell clone stably expressing si-1, as compared to non-transfected and to puromycin selected, control siRNA (si-1c) transfected Jurkat cells. Labeling with aPDI served as loading control.
(1.13 MB TIF)

Video S1 Accumulation of Hv1 and Nox2 in the wall of phagosomes in a neutrophil granulocyte. Pseudo color, 3D projection established from a series of 1 µm thin confocal slices taken along the Z axis (perpendicular to the plane of the fibronectin coated coverslip to which the cells are attached). Green denotes aHv1-N labeling (AF 488), while 7D5 (Nox2) labeling is presented in red (AF 568). Only the lower of the two neutrophils in contact displayed labeling with aHv1-N. The double labeled cell engulfed 3 zymosan particles (see also Fig. 4, third column in the manuscript). Zymosan particles can be recognized as labeling-free spherical structures inside the cell. Intense, overlapping clustering of the two signals (yellow) provides better outline of one of the particles. In the analysis only pixels with intensity at least the average intensity observed in experiments performed with control antibodies were included. Projection was performed to the brightest points and pixels are interpolated.
(0.18 MB MPG)

Video S2 Accumulation of Hv1 and Nox2 in the wall of a phagosome in a differentiated PLB-985 cell. Pseudo color, 3D projection calculated from a series of 1 µm thin confocal slices taken along the Z axis (perpendicular to the plane of the fibronectin coated coverslip to which the cell is attached). Green denotes aHv1-N labeling (AF 488), while 7D5 (Nox2) labeling is presented in red (AF 568). Engulfed zymosan particle can be recognized as labeling-free spherical structure on the cell periphery (see also Fig. 4, fourth column in the manuscript). Intense, overlapping clustering of the two signals can be observed in yellow. In the analysis only pixels with intensity at least two times the average intensity observed in experiments performed with control antibodies were included. Projection was performed to the brightest points, and pixels are interpolated.
(0.25 MB MPG)
