# Supplementary figures and images for: Molecular and Functional Characterization of Hv1 Proton Channel in Human Granulocytes
Source: PLoS One. 2010 Nov 23;5(11):e14081. doi: 10.1371/journal.pone.0014081 (PMC2990768; doi:10.1371/journal.pone.0014081)

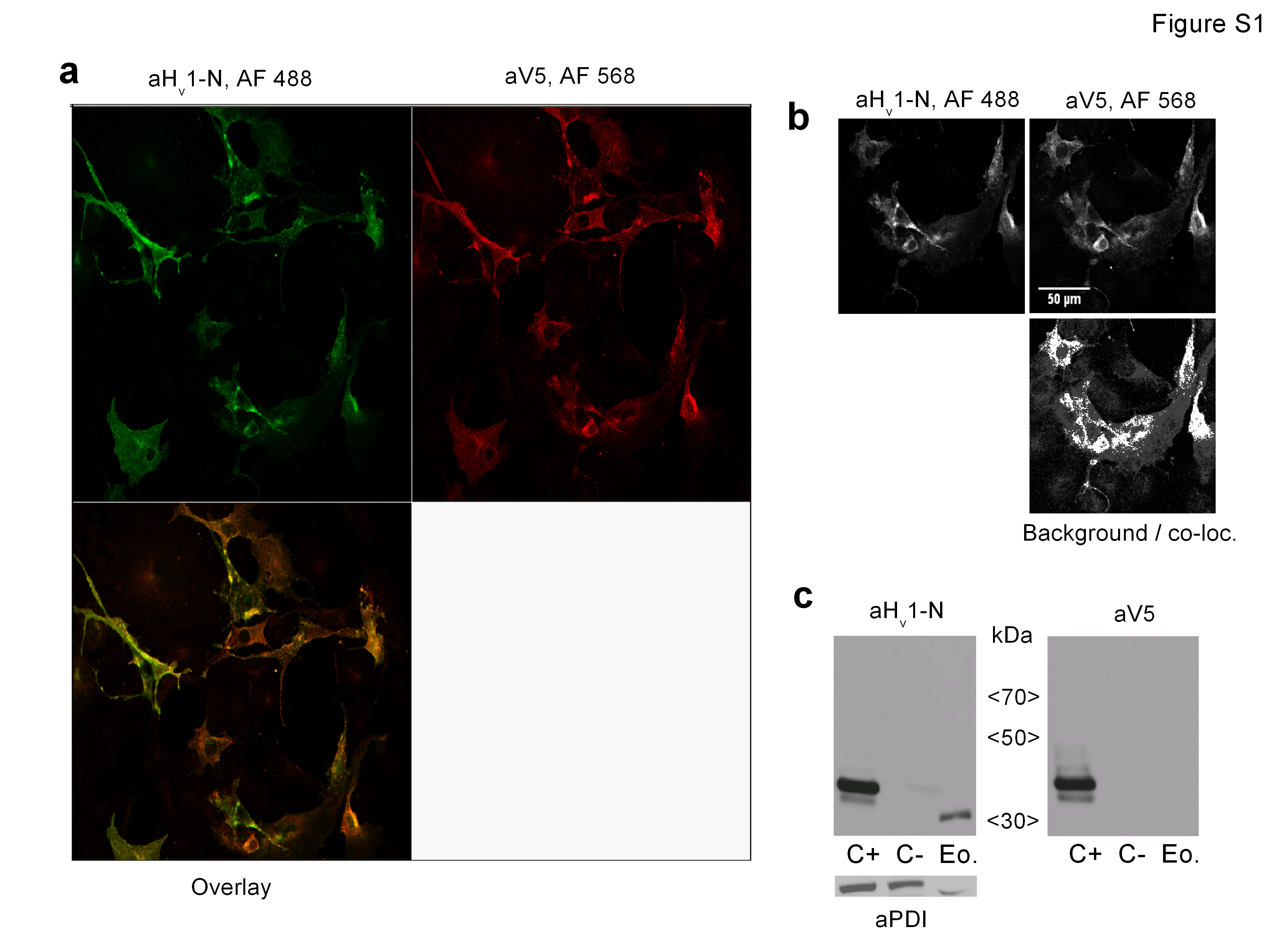

Supplement: Figure S1 — A polyclonal antibody selectively recognizes native Hv1. To test the specificity and sensitivity of the antibody, COS-7 cells were transiently transfected with plasmid encoding V5-epitope tagged full length Hv1 (Hv1-V5). (a) Detection of colocalization of anti-V5 and aHv1-N labeling in Hv1-V5 transfected COS-7 cells in immunofluorescence experiments using confocal laser scanning microscopy (Pearson's coefficient is 0.87). The two antibodies label the same cells and cellular structures. Secondary antibodies were labeled with Alexa Fluor® (AF, scale bar, 50 µM). In mock transfected cells only very weak auto fluorescence was detectable (not shown). (b) Result of colocalization analysis performed on pixels with above threshold intensities (see methods). Colocalizing pixels are displayed as white dots in the most right column. (c) Anti-V5 and aHv1-N both detect Hv1-V5 in COS-7 cells transfected with the construct (C+), but not in mock transfected (C-) cells. Only aHv1-N recognizes native Hv1 in human eosinophils (Eo.). Labeling the house keeping enzyme PDI with aPDI served as loading control. The estimated molecular weights (m.w.) of the Hv1 monomer and our Hv1-V5 construct are ∼31.6 and ∼36.5 kDa, respectively. (4.81 MB TIF) [file pone.0014081.s001.tif]

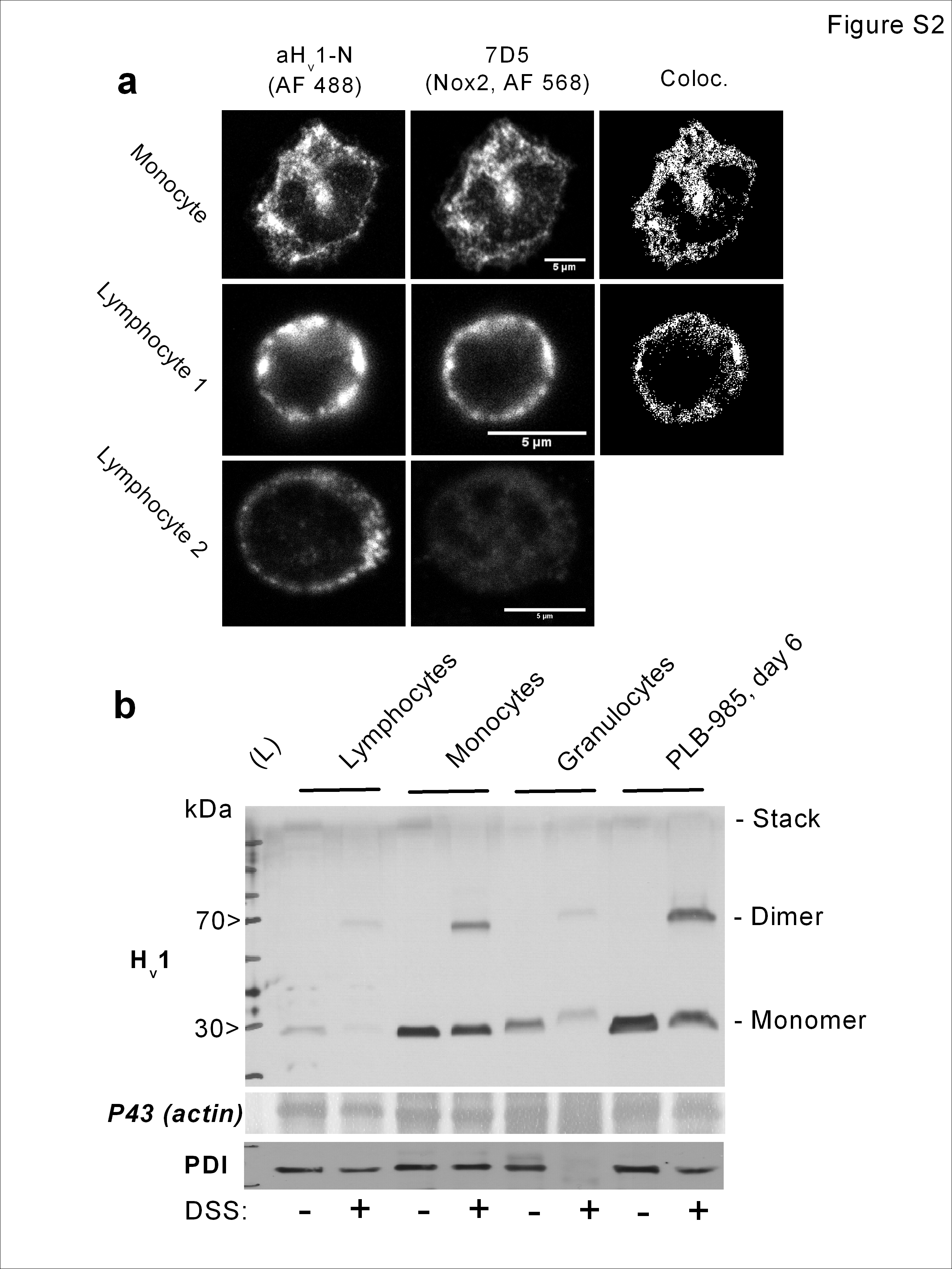

Supplement: Figure S2 — Localization and structure of Hv1 in human leukocytes. (a) Detection of Hv1 (most left column) and Nox2 (middle) in mononuclear cells. Colocalization analysis was performed only in cells in that at least moderate labeling (two times above background) for both proteins could be detected. Above threshold colocalizing pixels are displayed as white dots in the most right column. After correction for differences in mean signal intensities between Hv1 and Nox2 labeling, threshold was set to 20% of maximal detectable intensity. Monocytes displayed strong labeling with aHv1-N, sometimes with strong, colocalizing 7D5 signal. Based on their staining pattern, lymphocytes could be classified into two major groups. Cells in the first group were not stained with either of the two antibodies (not shown), while those in the other group showed strong aHv1-N labeling with strong (lymphocyte 1) to background (lymphocyte 2) 7D5 signal. In lymphocytes aHv1-N labeling often displayed a patchy pattern, which likely arises from endocytotic vesicles (as demonstrated earlier by others [Capasso M. et al., 2010, Nat Immunol 11: 265–272]). Pearson's coefficient for lymphocytes is 0.80±0.02 (n = 4). Scale bars represent 5 µm; Pearson's coefficient for the displayed monocyte is 0.92. (b) Demonstration of Hv1 dimers in human mononuclear leukocytes after cross-liking with DSS in WB after a standard reducing PAGE. Cells were preincubated with DFP (1∶5000), and DFP was present during crosslinking. (3.80 MB TIF) [file pone.0014081.s002.tif]

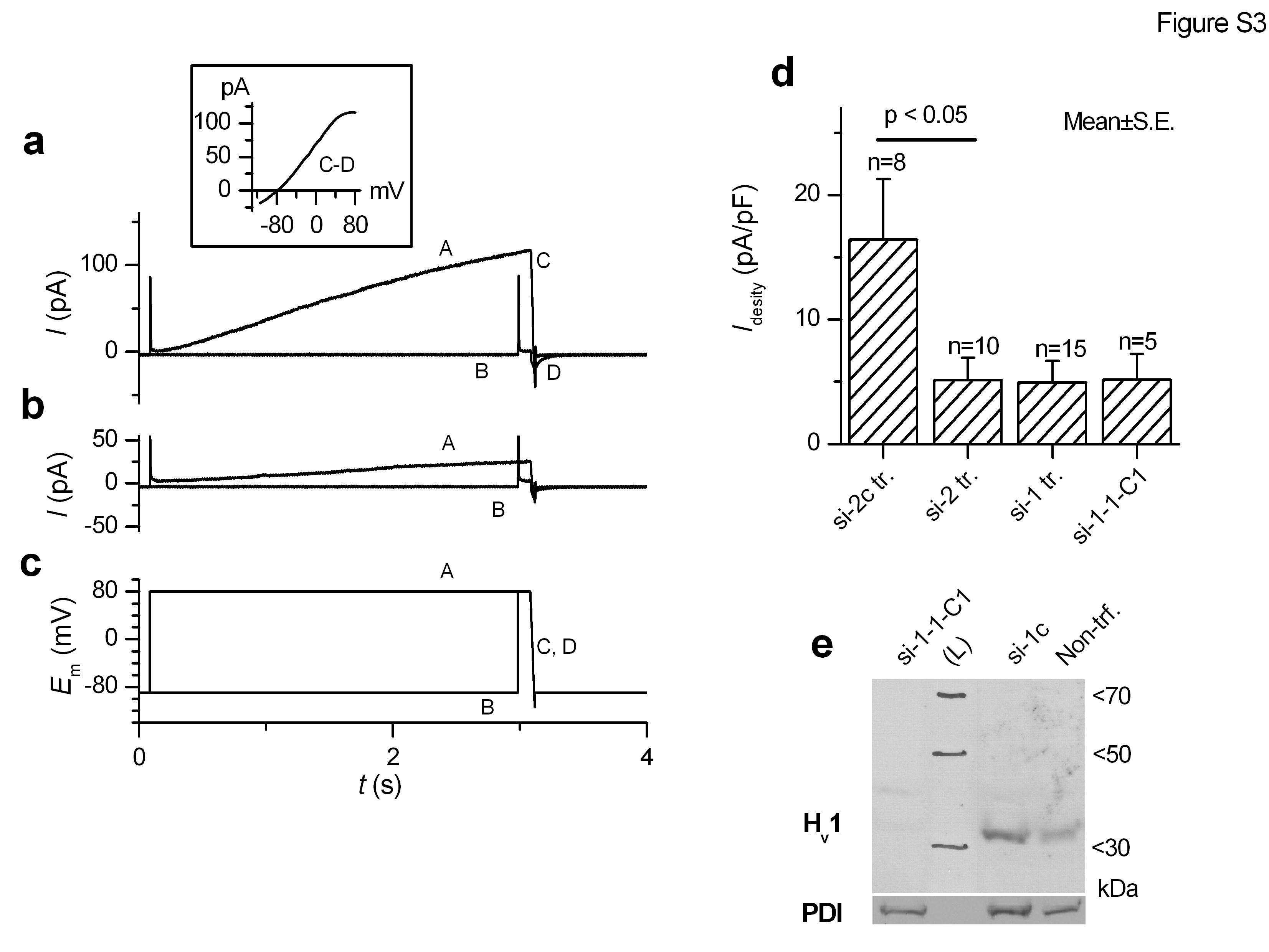

Supplement: Figure S3 — Hv1 supports voltage-gated proton current in the human lymphoid leukemia cell line Jurkat. (a) Representative curves from whole cell patch-clamp recordings demonstrate voltage-gated proton current in Jurkat cells transfected with control siRNA, si-2c. Every 15 s, a long-lasting (3 s, A) or short-lasting (0.2 s, B) activating pulse to 80 mV was applied, followed by a rapid voltage ramp to the -90-mV holding potential (C, D). The reversal potential of the voltage- and time-dependent current (inset) was determined by subtracting the two “ramp tail” currents (C–D). (b) Representative curves demonstrate voltage-gated proton current in Jurkat cells transfected with a siRNA (si-2) effective in knocking down Hv1 protein in Hv1-V5 transfected COS-7 cells (not shown). Recordings of transiently transfected Jurkat cells were performed 48–96 h after transfection. Transiently transfected Jurkat cells were identified based on their GFP fluorescence by illuminating the cells at 488±12 nm using a monochromator equipped 75 watt xenon arc lamp of a monochromator equipped fluorimeter (PTI, South Brunswick, NJ, USA). (c) Voltage protocols used in a and b. (d) Cumulative data of proton current density (amplitude of the time dependent current component normalized to whole cell capacitance) in Jurkat cells transfected transiently with control (si-2c) or effective (si-1 and si-2) siRNA constructs. Si-1-1-C1 is a puromycin selected clone stably expressing si-1. (e) Western blot detection of Hv1 in Jurkat cells. Expression level of the ∼36 kDa apparent m.w. protein is reduced in puromycin selected Jurkat cell clone stably expressing si-1, as compared to non-transfected and to puromycin selected, control siRNA (si-1c) transfected Jurkat cells. Labeling with aPDI served as loading control. (1.13 MB TIF) [file pone.0014081.s003.tif]
